# Mismatch repair, p53, and L1 cell adhesion molecule status influence the response to chemotherapy in advanced and recurrent endometrial cancer

**DOI:** 10.1186/s12885-024-13294-3

**Published:** 2024-12-30

**Authors:** Jung Chul Kim, Byungsoo Ahn, Yong Jae Lee, Eun Ji Nam, Sang Wun Kim, Sunghoon Kim, Young Tae Kim, Eunhyang Park, Jung-Yun Lee

**Affiliations:** 1 Department of Obstetrics and Gynecology, Institution of Women’s Medical Life Science, Yonsei University College of Medicine, 50-1 Yonsei-Ro, Seodaemun-Gu, Seoul, 03722 Korea; 2 Department of Pathology, Severance Hospital, Yonsei University College of Medicine, 50-1 Yonsei-Ro, Seodaemun-Gu, Seoul, 03722 Korea

**Keywords:** Endometrial neoplasms, Molecular classification, Neural cell adhesion molecule L1 (L1CAM), Prognosis, Recurrence, Survival

## Abstract

**Objective:**

This study aimed to identify the recurrence and survival rates according to the mismatch repair (MMR), p53, and L1 cell adhesion molecule (L1CAM) status in patients with advanced and recurrent endometrial cancer (EC) receiving systemic chemotherapy.

**Methods:**

This single-center retrospective cohort study included chemotherapy-naïve patients with advanced-stage (III/IV) or recurrent EC between January 2015 and June 2022 (*n* = 156), who were administered chemotherapy as adjuvant therapy or first-line palliative treatment. MMR and p53 status were assessed, and L1CAM was tested using immunohistochemistry in the p53-wild and MMR-proficient (p53wt/pMMR) group. The primary outcomes were progression-free survival (PFS) and overall survival (OS).

**Results:**

Of the 156 patients, 62 (39.7%), 53 (34.0%), and 41 (26.3%) had p53wt/pMMR, abnormal p53 (p53abn), and MMR-deficient (dMMR) tumors, respectively. PFS and OS were longest in dMMR, followed by p53wt/pMMR, and were the least in p53abn tumors (PFS: *p* = 0.0006, OS: *p* = 0.0013). After p53wt/pMMR was classified according to positive or negative L1CAM status, the L1CAM negative group exhibited significantly shorter survival rates than the L1CAM positive group (PFS: *p* = 0.0001, OS: *p* = 0.0027). p53abn tumors were independent prognostic factors for poor PFS (PFS: *p* = 0.039 on multivariable analysis).

**Conclusion:**

In chemotherapy-naïve patients with advanced and recurrent EC, there was a better prognosis in the order of MMR-D, p53wt/pMMR, and p53abn tumors after chemotherapy. L1CAM status is useful as a new marker to stratify p53wt/pMMR in advanced and recurrent groups.

**Supplementary information:**

The online version contains supplementary material available at 10.1186/s12885-024-13294-3.

## Introduction

Endometrial cancer (EC) is the sixth most common cancer in women. Annually, approximately 417,000 new patients are identified worldwide, and approximately 97,000 die [1]. There is also an increasing trend in Korea, with cases increasing from 727 in 1999 to 3,287 in 2019. This annual increase is likely due to increased exposure to endogenous and exogenous estrogens associated with risk factors such as obesity and diabetes and increased life expectancy [2, 3].

With the use of whole genome sequencing, The Cancer Genome Atlas (TCGA) research network has divided EC into four molecular subtypes: DNA polymerase epsilon (POLE)-mutated (ultramutated), microsatellite instability-high (MSI-H, hypermutated), copy-number low (CN low), and copy-number high (CN high), and the prognosis differs according to the subtype [4].

The subgroups could be more easily classified as mismatch repair (MMR) defective (dMMR, surrogate of MSI-H), POLE exonuclease domain mutant (POLE EDM, surrogate of POLE-mutated), p53 wild-type (p53wt), and p53 null/missense mutations (no specific molecular profile [NSMP] and p53 abnormal [p53abn], surrogate of CN low and CN high, respectively) using MMR proteins, p53 status as a surrogate marker using immunohistochemistry (IHC) and POLE exonuclease domain hotspot sequencing [5, 6]. Through this subgrouping, the European Society of Gynaecological Oncology has recently advanced in suggesting treatment protocols [7, 8].

However, despite these classifications and treatment guidelines, studies on prognosis according to molecular subtype using chemotherapy have not been conducted in chemotherapy-naïve patients with advanced and recurrent EC.

In addition, among many studies on new markers beyond the current classification, the importance of the L1CAM-a type L1 protein is emerging. Therefore, it is necessary to evaluate whether the new classification using this marker can be used for determining the prognosis of patients with advanced EC and recurrent EC requiring primary adjuvant therapy [9]. 

Hence, this study examined the prognosis of patients with advanced and recurrent EC who received chemotherapy as adjuvant or palliative therapy by investigating progression-free survival (PFS) and overall survival (OS) to determine whether L1CAM can be a useful prognostic marker for these patients.

## Materials and methods

This retrospective cohort study was conducted at the Severance Hospital of the Yonsei University Health System (YUHS, 4–2023-0263).

### Study population

Patients who met the following inclusion criteria over the period from January 2015 to June 2022 were identified: (1) advanced-stage (III/IV) and recurrent EC with first recurrence after diagnosis and chemotherapy-naïve patients, and (2) adjuvant therapy in stage III or palliative therapy in stage IV/recurrence patients.

### Immunohistochemistry

Formalin-fixed paraffin-embedded slices (4 μm) were deparaffinized and rehydrated with xylene and alcohol solutions. Immunostaining was performed using an automatic immunostaining instrument (Ventana Benchmark XT; Ventana Medical Systems, Tucson, AZ, USA) according to the manufacturer’s instructions. IHC for MMR proteins (MLH1, MSH2, MSH6, PMS2), p53, and L1CAM was performed using the following antibodies: MutL homolog 1 (MLH1, 1:50, BD Biosciences, San Jose, CA, USA), MutS homolog 2 (MSH2, 1:200, BD Biosciences), MutS homolog 6 (MSH6, 1:100, Cell Marque, Rocklin, CA, USA), PMS1 homolog 2 (PMS2, diluted 1:40, Cell Marque), p53 (clone DO-7; 1:300, Novocastra, Leica Biosystems, Newcastle Upon Tyne, UK), and L1CAM (clone UJ127.11; 1:1000, Sigma, MO, USA).

For MMR proteins, tumors were considered aberrant if tumor cells showed a complete absence of nuclear staining with a positive non-neoplastic internal control and intact if tumor cells exhibited nuclear positivity. For p53, weak focal positive staining was defined as the p53 wild-type pattern, and aberrant expression was classified into three patterns: overexpression (diffuse and strong nuclear staining of > 70% of tumor cell nuclei), a complete absence of expression (no staining), and cytoplasmic expression (cytoplasmic staining of tumor cells). L1CAM evaluation was performed for patients in the p53-wild and MMR proficient group (p53wt/pMMR). For L1CAM evaluation, the percentage of positive membrane staining in tumor cells was scored regardless of staining intensity, and tumors with ≥ 10% positivity were considered L1CAM positive, based on a previous study (Supplement 1. immunohistochemical images of L1CAM expression (positive and negative)) [10]. Two pathologists (B.A. and E.P.) reviewed all slides, blinded to the patient characteristics and outcomes. If discrepancies occurred, discussion ensued until a consensus was reached.

### Statistical analysis

Statistical analyses were performed using IBM SPSS statistics software (version 21.0; IBM Corp., Armonk, NY, USA) and Prism software (GraphPad, La Jolla, CA, USA). Clinical and demographic characteristics were compared among women using Fisher’s exact test for categorical data and the Wilcoxon rank-sum test for continuous data. The incidence rate of recurrence (recurrence rate) was calculated for each subgroup. Finally, we compared PFS and OS among the subgroups using Kaplan–Meier curves and log-rank tests. PFS was defined as the time between surgery and recurrence or death and was censored at the last follow-up visit, whereas OS was defined as the time between surgery and death and was censored at the latest follow-up visit. We used Cox proportional hazards regression to estimate the effect of molecular subtypes while adjusting for covariates for both PFS and OS among the subgroups. Clinical and demographic variables that were significant in the univariate analysis (*p* < 0.05) were included in the multivariable Cox model.

## Results

**Fig. 1:**
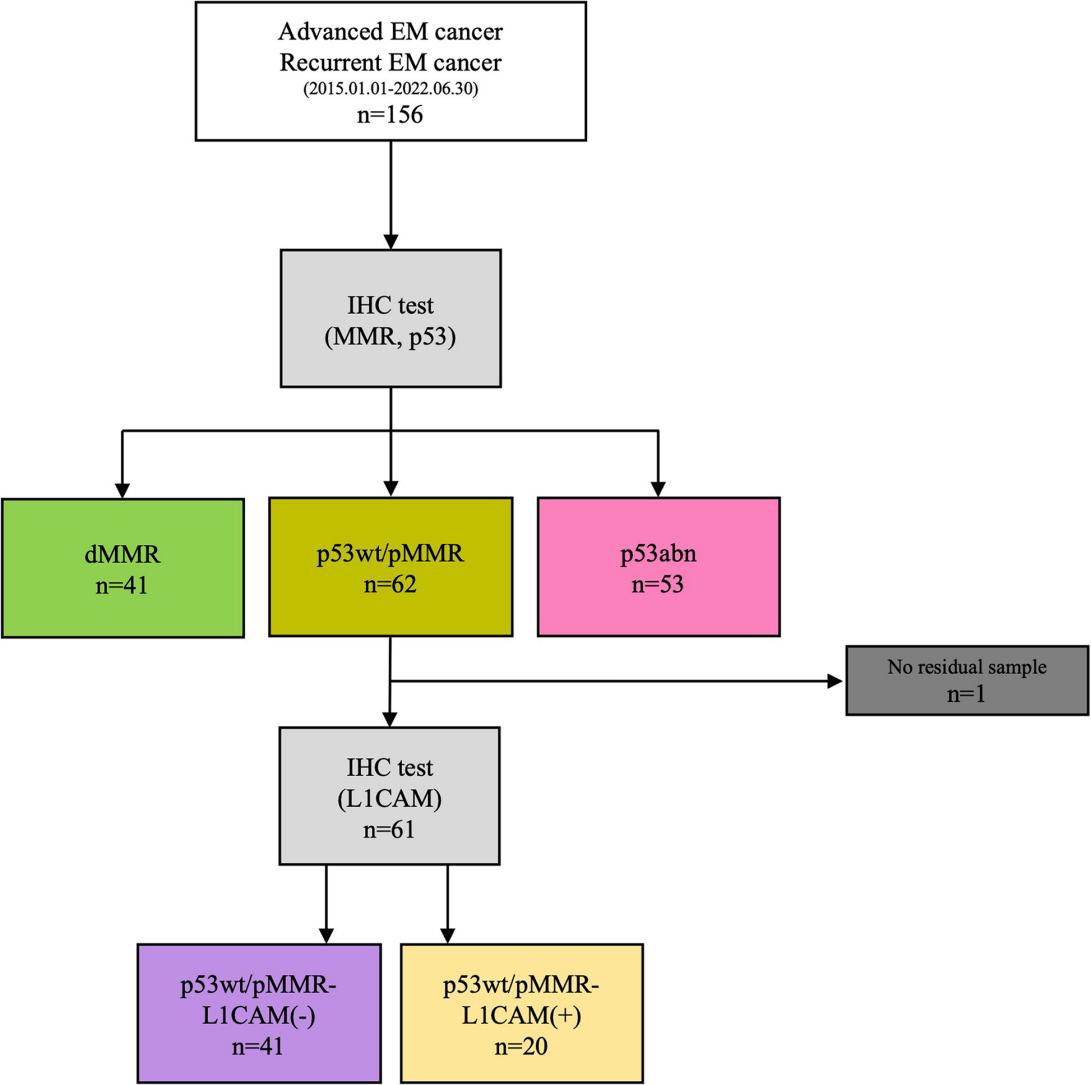
Trial profile. *Abbreviations: MMR, mismatch repair; dMMR, mismatch repair deficiency; p53wt/pMMR, p53-wild and MMR proficient group; p53abn, abnormal p53

The flow diagram of this study is presented in Fig. 1. Between January 2015 and June 2022, 156 patients with advanced-stage/recurrent EC identified at the Severance Hospital of YUHS had undergone MMR and p53 tests.

**Fig. 2:**
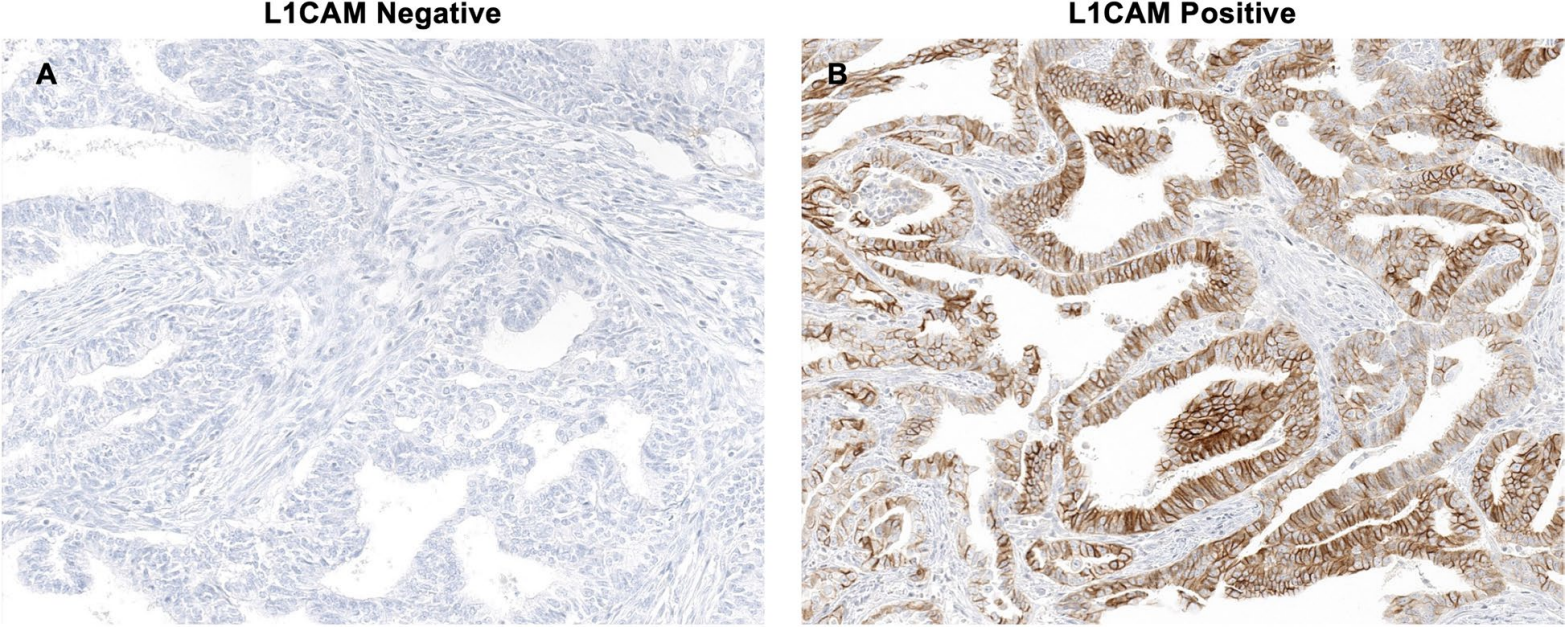
Representative images of L1CAM immunohistochemical staining. **A** L1CAM negative **B** L1CAM positive (20 × magnification)

A total of 41, 62, and 53 patients were identified in the dMMR, p53wt/pMMR, and p53abn groups, respectively (Fig. 1). Among them, the expression of L1CAM was confirmed using IHC in the p53wt/pMMR group. Of the 62 patients, 41 and 20 were identified as L1CAM negative and L1CAM positive, respectively, whereas one patient did not have a specimen for IHC testing (Fig. 2).

Assessment of the characteristics of the subgroups divided according to molecular classification indicated that women in the dMMR group were younger, while those in the p53abn group were older. Moreover, relatively lower advanced-stage (68.3% stage III) and predominantly endometrioid (90.2%) tumors were found in the dMMR group. The p53abn tumors displayed aggressive pathological features (73.6% grade 3, 49.1% nonendometrioid histology, 64.2% lymph-vascular space invasion [LVSI], and higher advanced stages). As the p53wt/pMMR group was divided based on L1CAM IHC, in the L1CAM-positive group, older age, more menopausal states, and aggressive pathologic features (50.0% grade 3, 25.0% nonendometrioid histology, 55.0% LVSI, and higher advanced stages) were identified compared with the L1CAM-negative group (Table 1).

**Table 1:** Demographic and clinical characteristics overall and by molecular classification and L1CAM status

	dMMR (*N* = 41)	MMR-proficient			Total (*N* = 155)	*p* value
		p53wt/pMMR -L1CAM(-) (*N* = 41)	p53wt/pMMR -L1CAM( +) (*N* = 20)	p53abn (*N* = 53)		
Age(years)						
Mean (SD)	51.7(± 9.5)	55.6(± 10.7)	60.1(± 9.4)	59.6(± 10.7)	56.5(± 10.7)	0.001
BMI						
Mean (SD)	23.7(± 4.5)	25.4(± 4.0)	23.5(± 3.9)	25.2(± 5.3)	24.6(± 4.6)	0.201
Parity						
0	17(41.5%)	11(26.8%)	6(30.0%)	10(18.9%)	44(28.4%)	0.117
1 or more	24(58.5%)	30(73.2%)	14(70.0%)	43(81.1%)	111(71.6%)	
Diabetes						
No	35(85.4%)	33(80.5%)	17(85.0%)	46(86.8%)	131(84.5%)	0.863
Yes	6(14.6%)	8(19.5%)	3(15.0%)	7(13.2%)	24(15.5%)	
Prior malignancies						
No	38(92.7%)	41(100.0%)	19(95.0%)	45(84.9%)	143(92.3%)	0.053
Yes	3(7.3%)	0(0.0%)	1(5.0%)	8(15.1%)	12(7.7%)	
CA-125 at diagnosis						
Mean (SD)	130.4(± 24.0)	95.5(± 184.3)	59.1(± 67.6)	154.0(± 307.2)	120.0(± 220.1)	0.338
Histology						
Endometrioid	37(90.2%)	34(82.9%)	15(75.0%)	27(50.9%)	113(72.9%)	0.002
Serous	0(0.0%)	2(4.9%)	1(5.0%)	10(18.9%)	13(8.4%)	
Clear cell	2(4.9%)	1(2.4%)	1(5.0%)	4(7.5%)	8(5.2%)	
MMMT	1(2.4%)	2(4.9%)	1(5.0%)	11(20.8%)	15(9.7%)	
Adenocarcinoma	0(0.0%)	2(4.9%)	2(10.0%)	1(1.9%)	5(3.2%)	
Neuroendocrine	1(2.4%)	0(0.0%)	0(0.0%)	0(0.0%)	1(0.6%)	
Stage at diagnosis						
III	28(68.3%)	27(65.9%)	12(60.0%)	28(52.8%)	95(61.3%)	0.457
IV	7(17.1%)	4(9.8%)	5(25.0%)	13(24.5%)	29(18.7%)	
Recur	6(14.6%)	10(24.4%)	3(15.0%)	12(22.6%)	31(20.0%)	
Staging op pathological grade						
1	7(17.1%)	12(29.3%)	3(15.0%)	0(0.0%)	22(14.2%)	< 0.001
2	24(58.5%)	19(46.3%)	6(30.0%)	14(26.4%)	63(40.6%)	
3	9(22.0%)	9(22.0%)	10(50.0%)	39(73.6%)	67(43.2%)	
none	1(2.4%)	1(2.4%)	1(5.0%)	0(0.0%)	3(1.9%)	
Staging op LVSI						
No	15(36.6%)	22(53.7%)	7(35.0%)	19(35.8%)	63(40.6%)	0.055
Yes	25(61.0%)	19(46.3%)	11(55.0%)	34(64.2%)	89(57.4%)	
Missing	1(2.4%)	0(0.0%)	2(10.0%)	0(0.0%)	3(1.9%)	
Radiotherapy						
No	16(39.0%)	27(65.9%)	11(55.0%)	34(64.2%)	88(56.8%)	0.049
Yes	25(61.0%)	14(34.1%)	9(45.0%)	19(35.8%)	67(43.2%)	

*Recur* Recurrent endometrial cancer, *dMMR* Mismatch repair protein deficiency, *p53wt/pMMR* p53-wild with proficient mismatch repair, *p53abn* abnormal p53, *BMI* Body mass index, *MMMT* Malignant mixed Müllerian tumour, *LVSI* Lymph-vascular space invasion

**Fig. 3:**
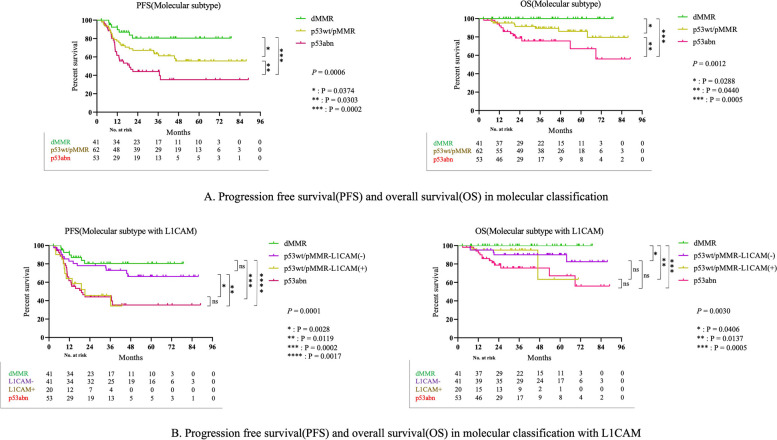
**A** Progression free survival (PFS) and overall survival (OS) in molecular classification. **B** Progression free survival (PFS) and overall survival (OS) in molecular classification with L1CAM

In the overall cohort, PFS and OS were longer in the order of dMMR, p53wt/pMMR, and p53abn tumors (Fig. 3**-A;** PFS: *p* = 0.0006, OS: *p* = 0.0013). After p53wt/pMMR classification according to L1CAM status, the L1CAM-negative group exhibited longer PFS and OS than the L1CAM-positive group (Fig. 3**-B;** PFS: *p* = 0.0001 and OS: *p* = 0.0030).

In addition, when PFS and OS were compared between patients with stage III disease who received adjuvant therapy and those with stage IV or recurrent disease who received palliative treatment, significant differences in PFS were confirmed based on the molecular subtype and L1CAM status in both groups (Supplement 1-A; PFS: *p* = 0.0205 in molecular subtype and PFS: *p* = 0.0018 in molecular subtype with L1CAM, Supplement 1-B; PFS: *p* = 0.0045 in molecular subtype and PFS: *p* = 0.0097 in molecular subtype with L1CAM). In addition, there was a significant difference in OS, except for the molecular subtypes with L1CAM in the adjuvant therapy group; there was also a difference in the overall tendency (Supplement 2-A; OS: *p* = 0.0236 in molecular subtype and OS: *p* = 0.0577 in molecular subtype with L1CAM**, **Supplement 2-B; OS: *p* = 0.0325 in molecular subtype and OS: *p* = 0.1977 in molecular subtype with L1CAM).

In multivariate analysis performed based on variables with significant *p* values in univariate analysis (Table 2), age, stage, and molecular classification exhibited a trend toward significance for PFS (*p* = 0.010, *p* = 0.009, and *p* = 0.039, respectively). Body mass index (BMI) was significantly associated with OS (*p* = 0.018). For PFS, the hazard ratios (HRs) for stage IV and recurrent EC regarding stage III were 2.137 (CI: 1.098–4.160) and 2.335 (CI: 1.240–4.398), respectively. The HRs for dMMR and p53abn regarding p53wt/pMMR were 0.579 (CI: 0.258–1.300) and 1.599 (CI: 0.911–2.806), respectively. Additionally, the HR for age was 1.036 (CI: 1.009–1.064), while the HR for BMI was 0.866 (CI: 0.768–0.976) for OS.

**Table 2:** Multivariable Survival Analysis in the Confirmation Cohort (*n* = 156) Using Parameters Available at the Time of Diagnosis

	PFS		OS	
Variable [Ref]	HR (95% CI)	*p* value	HR (95% CI)	*p* value
Age	1.036(1.009–1.064)	0.010		
Stage [III]		0.009		
IV	2.137(1.098–4.160)			
Recur	2.335(1.240–4.398)			
CA-125	1.001(1.000–1.002)	0.061		
BMI			0.866(0.768–0.976)	0.018
Radiotherapy[No]				0.074
Yes			0.367(0.122–1.101)	
Molecular classification [p53wt/pMMR]		0.039		0.172
dMMR	0.579(0.258–1.300)		0.000(0.000–5.407E173)	
p53abn	1.599(0.911–2.806)		2.430(0.960–6.151)	

*HR* Hazard ratio, *Recur* recurrent endometrial cancer, *dMMR* mismatch repair protein deficiency, *p53wt/pMMR* p53-wild with proficient mismatch repair, *p53abn* abnormal p53, *BMI* body mass index

## Discussion

### Summary of main results

We confirmed that PFS and OS were the worst for the p53abn subtype (p53abn < p53wt/pMMR < dMMR) of advanced/recurrent EC after the first-line chemotherapy. This study compared PFS and OS after chemotherapy in patients with advanced or first recurrent EC. Therefore, it is valuable as a predictor for chemotherapy-naïve patients undergoing corresponding treatment. Additionally, we found differences in PFS and OS in the p53wt/pMMR group in patients with advanced-stage/recurrent EC using L1CAM IHC results. This study’s multivariable analysis revealed that age, stage, and molecular classification were associated with PFS and that BMI was associated with OS.

### Results in the context of published literature

In the Ruby trial, which compared the difference between platinum-based chemotherapy and chemotherapy with dostarlimab according to MMR status in advanced/recurrent EC, the dMMR and pMMR groups that underwent platinum-based chemotherapy had 24-month PFS rates of 15.7% and 18.8%, respectively [11]. In contrast, in the GY-018 trial comparing the difference between platinum-based chemotherapy and chemotherapy with pembrolizumab according to MMR status in advanced/recurrent EC, the dMMR with chemotherapy group exhibited a better prognosis than the pMMR with chemotherapy group [12]. This difference appears to depend on the patients who participated in the studies. In the present study, 43.59% of all patients and 61.0% of the dMMR group received radiotherapy, compared to 18.1% of all patients and 20% of the dMMR group in the Ruby trial, justifying the differences in the results. Additionally, in the GY-018 trial, 42.7% in the dMMR group and 39.6% in the pMMR group received radiotherapy, suggesting that there may be differences depending on radiotherapy treatment [11, 12]. In the PORTEC-3 study, the 5-year failure-free survival rates of patients with stage III EC treated with radiotherapy and chemoradiotherapy were reported to be 58.4% and 70.9%, respectively, which appears to indirectly explain the increase in PFS in this study [13].

In the NRG/GOG0210 study and as reported by Kim et al., whereby adjuvant therapy was administered to dMMR and pMMR patients in all stages of EC, the difference between both groups was affected by another risk factor that was confirmed in the univariate and multivariate analyses [14, 15]. These risk factors guide treatment decisions, as suggested in various guidelines. In the current ESGO/ESTRO/ESP guidelines published in 2020, the molecular classification, which shows the difference between PFS and OS, was integrated to form a new classification. Risk factors (histopathological type, grade, myometrial invasion, LVSI, etc.) were combined to classify the risk groups as low, intermediate, high-intermediate, high, and advanced metastatic. However, despite the bias of these risk factors, this study confirmed a clear difference in each molecular classification group using chemotherapy. There are implications that molecular classification can be used as a predictor to evaluate the prognosis of patients after chemotherapy [7, 16].

Recently, various studies have been conducted on additional markers with characteristics other than those of the four existing molecular classifications [17]. Kommos et al. suggested that L1CAM is a risk factor for stratifying patients with NSMP. L1CAM (CD171) is known to be closely associated with the epithelial-to-mesenchymal transition as a substance related to tumor cell motility and showed a significant difference between specific survival and OS in EC [9]. The subgroup of NSMP-L1CAM-positive tumors associated with high histological grade and high International Federation of Gynecology and Obstetrics stage had as poor an outcome as p53abn tumors [9, 17]. Based on the study of L1CAM in EC, the PORTEC-4a study is underway to confirm the results of adjuvant radiotherapy by newly defining favorable, intermediate, and unfavorable groups through molecular classification and additional L1CAM and CTNNB1 results as new markers in patients with early-stage EC [18]. In the present study, L1CAM was utilized as a novel marker to stratify patients with endometrial cancer (EC) and was anticipated to serve as a predictive marker following adjuvant treatments, such as chemotherapy. Furthermore, various studies have highlighted the role of L1CAM in endometrial cancer, fostering significant expectations for its potential clinical applications (Table 3).

**Table 3:** Recent studies on L1CAM of endometrial cancer

Title (Year)	Author	Objective	Major Finding
The role of L1CAM as predictor of poor prognosis in stage I endometrial cancer: a systematic review and meta-analysis (2023)	Giannini, A. et al	To evaluate the prognostic significance of L1CAM expression in patients with stage I endometrial cancer	High L1CAM expression was associated with worse DFS and OS. Additionally, high L1CAM levels correlated with more aggressive FIGO grade and older age
Enhanced Risk Stratification in Early-Stage Endometrial Cancer: Integrating POLE through Droplet Digital PCR and L1CAM (2023)	Joe, S. et al	To enhance risk stratification in early-stage endometrial cancer by integrating POLE mutations via ddPCR and L1CAM expression	L1CAM overexpression, especially in the NSMP subgroup, was significantly associated with poorer prognosis. POLE ddPCR identified mutations cost-effectively, showing potential as an alternative to NGS
Prognostic significance of L1CAM expression in addition to ProMisE in endometrial cancer (2023)	Kim, J. et al	To evaluate the prognostic value of L1CAM expression in conjunction with the ProMisE molecular classification for endometrial cancer	L1CAM was identified as an independent poor prognostic factor for PFS, especially in p53 wild-type cases, whereas β-catenin and PD-L1 were not associated with recurrence
Evaluation of prognostic potential of β-catenin and L1CAM expression according to endometrial cancer risk group (2024)	Yoon, H. et al	To assess the prognostic roles of β-catenin and L1CAM expressions in endometrial cancer risk groups	β-catenin was associated with poor PFS in high-intermediate-risk patients, while L1CAM was linked with advanced pathological features but was not an independent prognostic factor
Prognostic Significance of L1 Cell Adhesion Molecule in NSMP Endometrial Cancer (2024)	Ozdemir, C.Y. et al	To investigate the role of L1CAM expression in endometrial cancers classified as having NSMP for improving prognostic stratification	L1CAM positivity was significantly associated with worse outcomes, including higher recurrence rates, lymphovascular invasion, and lower progression-free and overall survival, especially within the NSMP group

*L1CAM* L1 Cell Adhesion Molecule, *DFS* disease-free survival, *OS* overall survival, *ddPCR* droplet digital PCR, *IHC* immunohistochemistry, *NGS* Next Generation Sequencing, *PFS* progression-free survival, *dMMR* Mismatch repair-deficient, *p53abn* p53 abnormal, *NSMP* no specific molecular profile

Various studies have demonstrated that the p53abn subtype exhibits a poor prognosis compared with that for other subtypes, and there are no treatment options other than chemotherapy. In this study, the prognosis of this group was poorer than that of the other groups. Samarnthai et al. observed that TP53 mutations are more common in type II EC, which is generally known to progress rapidly and has a poorer prognosis than type 1 EC [19]. Moreover, TP53 mutations can be identified through abnormal p53 IHC results, such as complete absence, overexpression, and cytoplasmic expression of p53 [20]. If proper repair of DNA damage is not achieved, apoptosis proceeds through apoptosis-signaling genes, such as *BAX*, *PUMA*, *Nox*, and *PERP*. Since carcinogenesis can occur when there is a functional abnormality in p53, the inability of this repair mechanism can be considered the potential underlying mechanism reflecting the poor prognosis of the p53abn subtype [21].

Conversely, in the case of the dMMR subtype, as a highly immunogenic tumor, it induces the upregulation of tumor-infiltrating lymphocytes by producing high levels of tumor mutant antigen. Through this, the cell-mediated antitumor response increases, which appears to be related to prolonged survival [22].

Recently, based on the similar molecular profiles of p53abn and high-grade serous ovarian cancer (HGSOC) using TCGA genomic analysis [4], the development of a treatment protocol using poly (ADP-ribose) polymerase inhibitors has been considered because HGSOC possesses the characteristics of homologous recombination deficiency [22].

Additional classification using new markers, such as L1CAM, is expected to offer various treatment options. Chung et al. demonstrated that using progestin in dMMR patients yielded a poor response, evident from the lower complete or partial remission rates in terms of the best overall response in early EC compared to that in other molecular classification groups. Therefore, molecular classification could be used as an indicator to determine EC treatment options for women of childbearing age who need fertility preservation [23]. Although L1CAM was useful as a classification marker in the p53wt/pMMR group in this study, if molecular classification is refined through additional research, it is expected to provide other treatment options, such as fertility preservation in patients with EC.

Since the development of the Proactive Molecular Risk Classifier for Endometrial Cancer (ProMisE) molecular classification, classification methods using new diagnostic methods besides IHC have garnered attention. One method involves using cervical swab-based genomic DNA (gDNA) of EC through the conventional Pap smear technique [24]. The research team verified the loss of MSH2 or MSH6 and aberrant p53 expression using cervical swab-based gDNA and confirmed its value as a tool that can be used to layer ProMisE molecular classification based on tests and stratification. Although only the IHC test technique was used in our study, we expect that rapid treatment of the patient and subsequent treatment plan determination will be possible through the noninvasive early diagnosis and classification of EC.

The TCGA Research Network reported that the POLE-mutated subtype was identified in 7% of all endometrial cancers. Therefore, even in this study, there was a limitation in that it was difficult to exclude the possibility that the corresponding group exists in another molecular subtype [4]. Currently, there is no method for confirming POLE mutations using IHC. However, a technology that can detect POLE mutations using Droplet Digital PCR (ddPCR) developed recently has reached the commercialization stage. This test was designed to detect POLE mutations in exons 9 (P286R, S297F), 13 (V411L), and 14 (A456P, S459F). Mutations can be confirmed using ddPCR, which exhibits higher specificity and sensitivity than real-time PCR [25]. Furthermore, since the expression of the POLE mutation subtype is low in advanced/recurrent EC, it is unlikely to affect the results of this study significantly [26]. In the future, if the prognosis and characteristics of patients by molecular subtype are more clearly confirmed using this technology, it will be useful in guiding treatment protocols.

### Strengths and weaknesses

The strength of this paper is that it investigated molecular subtypes only for chemotherapy-naïve advanced/recurrent endometrial cancer. It is significant that through this, it was possible to evaluate the effect of chemotherapy more objectively and to classify the progression of the disease after chemotherapy by molecular subtype. In addition, it can be considered an important result that L1CAM, a new marker, is an indicator that can divide the prognosis from initial treatment for the p53wt/pMMR subgroup among advanced/recurrent endometrial cancers.

However there were a few limitations of this study. First, the POLE mutation subgroup was not divided due to the financial constraints in Korea, it was not feasible to apply NGS or ddPCR to all patient groups. Additionally, although the number is not large in all endometrial cancer patients, it is seen as a limitation that it cannot be investigated in more detail. The median follow-up interval was short (38.4 month), and therefore we may not have captured all recurrences and deaths. Also, immunotherapy was excluded from consideration, as it is not approved as a first-line treatment under the Korean insurance system during the period of this study.

### Implications for practice and future research

After chemotherapy for chemotherapy-naïve patients with advanced and recurrent EC, different PFS and OS are shown according to subgroups, and the IHC method, which is relatively clinically accessible, subdivides the p53wt/pMMR subgroup through L1CAM. Therefore, it is necessary to consider the follow-up period and treatment option based on the expected recurrence and mortality rates.

## Conclusion

In chemotherapy-naïve patients with advanced and recurrent EC, favorable PFS and OS were observed after chemotherapy in the order of dMMR, p53wt/pMMR, and p53abn tumors, and the L1CAM status in the p53wt/pMMR subtype showed a difference regarding PFS and OS.

## Supplementary information

Supplementary Material 1

Supplementary Material 2

## Data Availability

The datasets generated during and analyzed during the current study are not publicly available due to protect patient privacy but are available from the corresponding author on reasonable request.
