# Supplementary figures and images for: Mismatch repair, p53, and L1 cell adhesion molecule status influence the response to chemotherapy in advanced and recurrent endometrial cancer
Source: BMC Cancer. 2024 Dec 30;24:1586. doi: 10.1186/s12885-024-13294-3 (PMC11684106; doi:10.1186/s12885-024-13294-3)

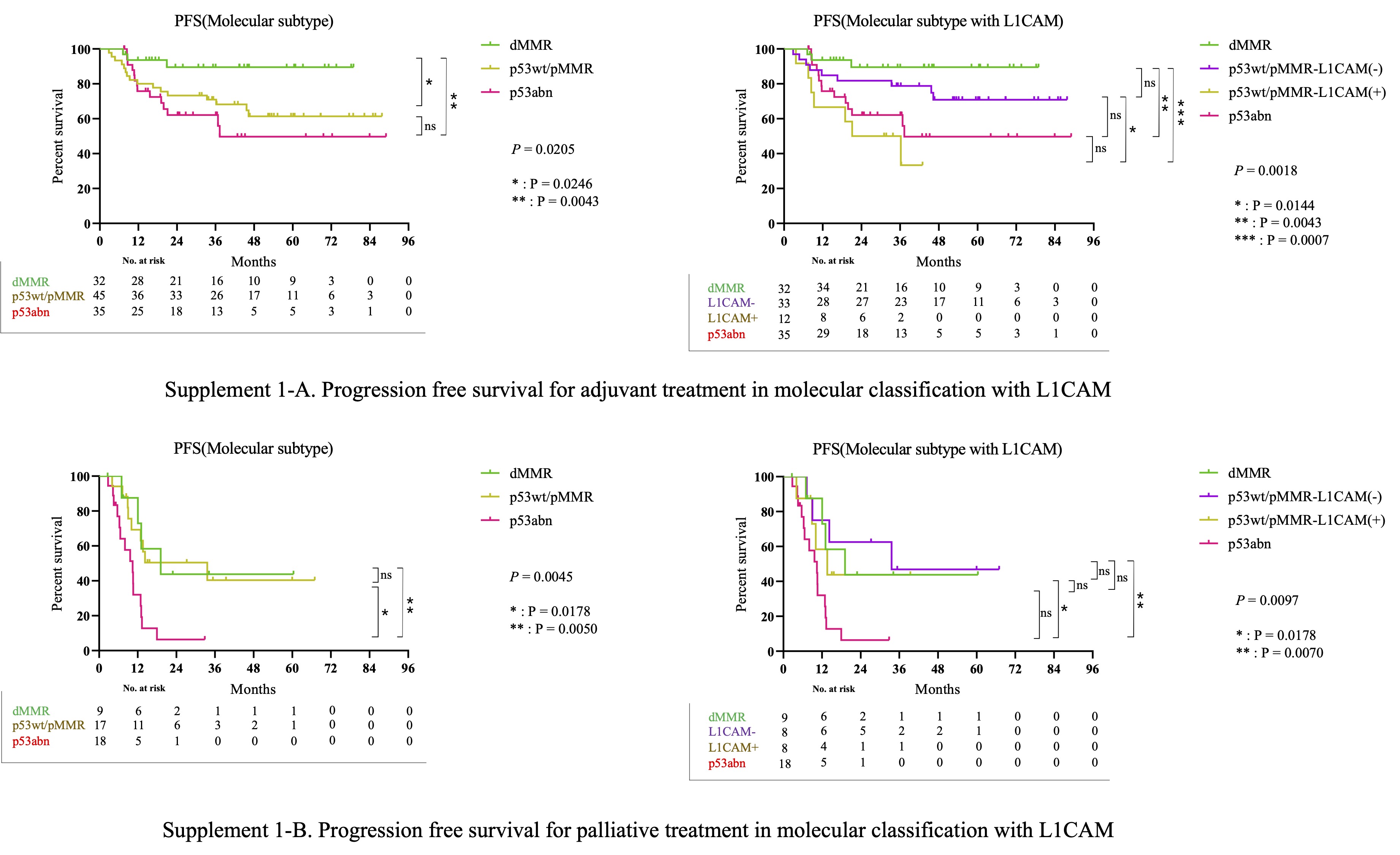

Supplement: Supplementary file 1 — Supplementary Material 1 [file 12885_2024_13294_MOESM1_ESM.jpg]

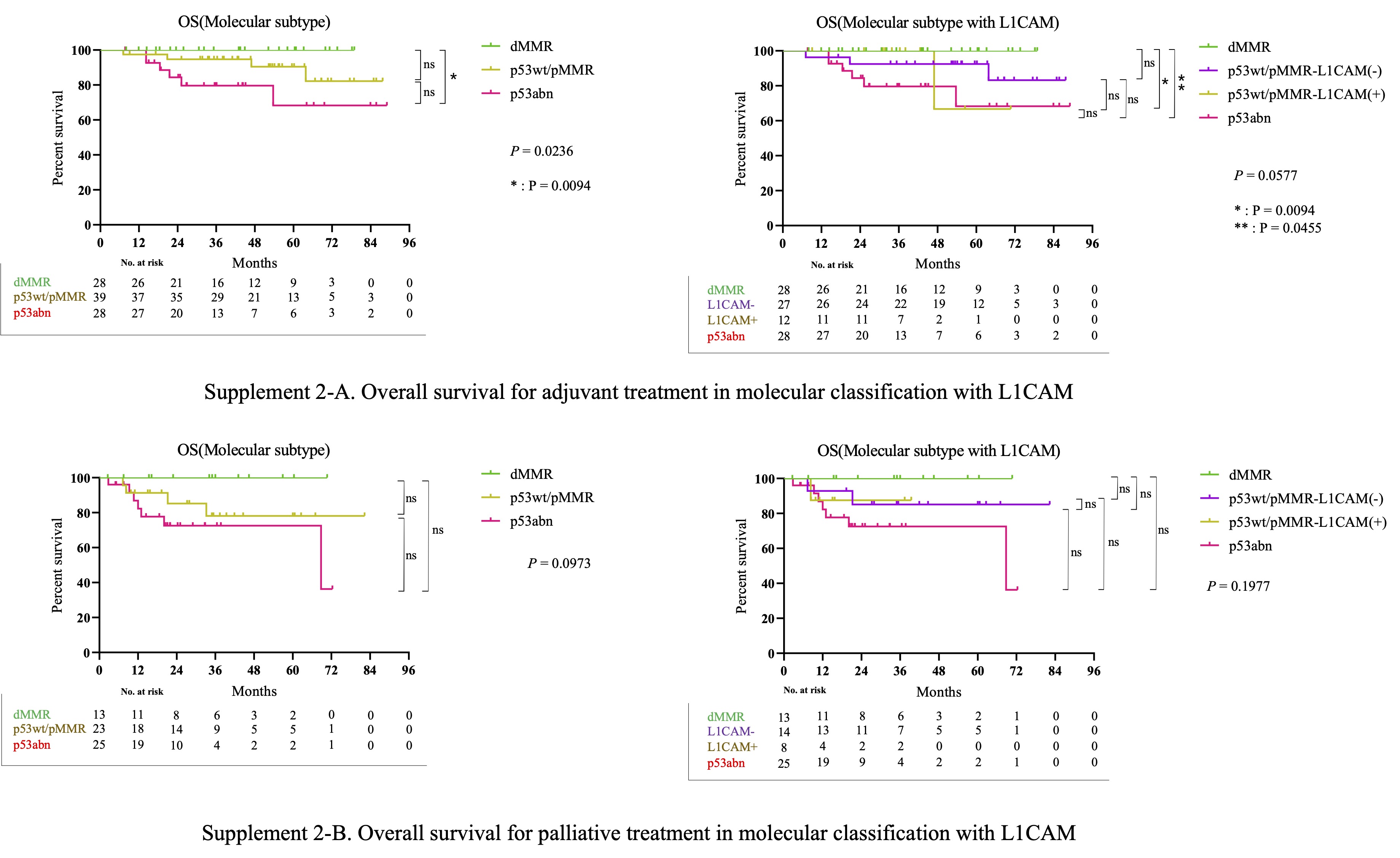

Supplement: Supplementary file 2 — Supplementary Material 2 [file 12885_2024_13294_MOESM2_ESM.jpg]
